# Chemical Composition and Vasorelaxant and Antispasmodic Effects of Essential Oil from *Rosa indica* L. Petals

**DOI:** 10.1155/2015/279247

**Published:** 2015-08-19

**Authors:** Hafiz Majid Rasheed, Taous Khan, Fazli Wahid, Rasool Khan, Abdul Jabbar Shah

**Affiliations:** ^1^Department of Pharmacy, COMSATS Institute of Information Technology, Abbottabad 22060, Pakistan; ^2^Biotechnology Program, Department of Environmental Sciences, COMSATS Institute of Information Technology, Abbottabad 22060, Pakistan; ^3^Institute of Chemical Sciences, University of Peshawar, Peshawar 25120, Pakistan

## Abstract

*Rosa indica* L. belongs to the family Rosaceae and is locally known as gulaab. It has different traditional uses in cardiovascular and gastrointestinal disorders but there is no scientific data available in this regard. Therefore, the basic aim of this study was to explore the chemical composition and gastrointestinal and cardiovascular effects of the essential oil obtained from *R. indica*. The chemical composition of the essential oil was investigated using gas chromatography-mass spectrometry (GC-MS) technique. The cardiovascular and gastrointestinal effects were investigated using electrophysiological measurements. The GC-MS analysis of the essential oil showed various chemical components including acetic acid, mercaptohexyl ester, butanoic acid, 2-methyl-5-oxo-1-cyclopentene-1-yl ester, artemiseole, methyl santonilate, isosteviol, caryophyllene oxide, pentyl phenyl acetate, dihydromyrcene, 1,5-octadecadien, octadecanoic acid, ethyl ester, palmitic acid (2-phenyl-1,3-dioxolan-4-yl methyl ester), santolina epoxide, and 9-farnesene. The electrophysiological measurements revealed that essential oil was more potent against K^+^ (80 mM) than phenylephrine precontractions using isolated rabbit aorta preparations. In isolated rabbit jejunum preparations, it showed more potency against high K^+^ induced contractions than spontaneous contractions. Considering these evidences, it can be concluded that *R. indica* essential oil may work as a complementary and alternative medicine in gastrointestinal and cardiovascular diseases.

## 1. Introduction

*Rosa indica* L. is a perennial flower shrub of the genus* Rosa*. It belongs to Rosaceae family, which contains herbs, shrubs, or trees that are rhizomatous, thorny, or climbing [1, 2]. Rosaceae is the 19th largest family and there are about hundred genera which are distributed from cosmopolitan to subcosmopolitan and diversified to northern hemisphere [2]. The leaves of rose are alternate and pinnately compound. They are sharply toothed oval-shaped leaflets. The fruit of plant is fleshy edible (rose hip) which ripens in the late summer (http://sindhforests.gov.pk/admin/MediaLibrary/Gulaab.pdf). The main areas of rose cultivation in Pakistan are Kallar Kahar, Choa SaidanShah, Chakwal, Islamabad, Pattoki, Faisalabad, and Sargodha. Some species of* Rosa* producing essential oil are also being cultivated in Sindh province [3]. The plants of this family are mainly grown for their beauty and fragrance. However, roses are a rich source of vitamin C and are used for making various medicinal herbal preparations [3]. Ethnopharmacologically, roses have been used in various eye diseases and heart disorders [4].

In the Indian system of medicine, various rose preparations are used as an astringent, tonic, mild laxative, and antibacterial agent and in treatment of sore throat, enlarged tonsils, and gall stones, for cooling effect, and as a vehicle for other medicines [5].* R. damascena* essential oil is believed to have pain killing and spasm relieving activities [6]. In addition, antimicrobial, anti-HIV, and hypnotic properties have also been reported for rose extract and its isolates [7, 8]. It was also observed that, when taken as food, rose oil has positive impact on various digestive tract disorders [9].

In a previous study, quinic acid (43.12%), 5-hydroxymethylfurfural (11.52%), pyrogallol (21.92%), levoglucosan (5.69%), and 4H-pyran-4-one,2,3-dihydro-3,5-dihydroxy-6-methyl (8.31%) were the major identified components in methanolic extract of* R. indica* petals [10]. Likewise, another study showed that volatile oils of fresh flowers of* R. damascena* mainly have citronellol, geraniol, nonadecane, and heneicosane in the essential oil, while they have alcoholic components (66.2%–80.7%), citronellol (1.8%–5.5%), and geraniol (3.3%–7.9%) in rose water fraction [11]. There was no scientific information available for the vascular and gastrointestinal effects of rose petals essential oil. Therefore, based on the above mentioned ethnomedicinal uses and other scientific information, this study was carried out to assess the potential of* R. indica* essential oil as an alternative treatment for vascular and gastrointestinal diseases. The results provided evidences that essential oil of rose petals may have positive effects on cardiovascular and gastrointestinal disorders.

## 2. Materials and Methods

### 2.1. Standard Drugs and Chemicals

Acetylcholine perchlorate, sodium chloride, magnesium sulphate, dihydrogen potassium phosphate, potassium chloride, magnesium chloride, sodium dihydrogen phosphate, sodium bicarbonate, glucose, TWEEN 80, DMSO (dimethyl sulfoxide), EDTA (Ethylenediaminetetraacetic Acid) and verapamil hydrochloride were the main chemicals and standard drugs used in the current study. All of these chemicals were of analytical grade and purchased from Sigma Chemicals Co. (St. Louis, MO, USA). Sodium sulphate and calcium chloride were attained from Merck (Darmstadt, Germany). Diethyl ether was purchased from Reanal Fine Chemicals Co. (Hungary) while chloroform was acquired from Lab-Scan Company Ltd. (Bangkok, Thailand).

### 2.2. Collection and Identification of* R. indica*

The fresh petals of* R. indica* were collected from the rose garden of Agriculture University of Peshawar. Professor Dr. Shazia Anjum, Director of Cholistan Institute of Desert Studies (CIDS), Islamia University, Bahawalpur, authenticated the collected plant materials. The voucher specimen of the plant (3519/CIDS/IUB) was deposited in the herbarium of CIDS.

### 2.3. Distillation of Plant Material

Steam distillation method was used to obtain essential oil from the fresh petals of* R. indica*. In this method, generator was used to produce steam, which was passed through the fresh rose (*R. indica*) petals containing flask through a glass pipe. The vapors were formed in the flask and passed through the neck to condenser. These vapors were condensed and collected in a receiver as a distillate. The distillate was kept in an amber colored and well closed glass container and stored at 4–8°C.

### 2.4. Essential Oil Separation

The steam distillate was added with diethyl ether to separate oily layer from aqueous layer. The mixture was placed in the separating funnel, shaken well for 30 min, and allowed to stand for 1 h to settle down the aqueous layer. The upper organic layer contained the essential oil and diethyl ether. Sodium sulphate was used as desiccant to remove water traces from organic layer. Solvent was evaporated at a low temperature in order to obtain pure essential oil.

### 2.5. Chemical Composition of the Essential Oil

In order to determine the major chemical constituents the obtained essential oil was evaluated using gas chromatography-mass spectrometry (GC-MS). The gas chromatographic analysis was performed on Clarus-600, Elite-5 MS model (Perkin Elmer Company, USA) equipped with 250 *μ*m column (diameter) with 30 m length. Clarus-600-C was used as an electron ionization detector. The GC condition was set as follows: injector temperature, 250°C; carrier gas, helium; and flow rate, 1 mL/min. The starting oven temperature was 50°C and then increased at the rate of 10°C/min up to 300°C (end temperature). The sample was dissolved in chloroform and injected onto the column. Mass spectrometry condition was set as voltage 70 eV, and mass scan range was 0–450 atomic mass units. The obtained spectrum was compared with the fragmentation pattern available with the NIST Library [12].

### 2.6. Animals

In the current study rabbits of either sex weighing 1–1.5 kg were used as a model animal. Animals were kept and cared for in the animal house of COMSATS Institute of Information Technology (CIIT), Abbottabad, according to the rules of ethical committee of CIIT, which completely agreed with the recommendations of the Institute of Laboratory Animal Resources, Commission on Life Sciences, National Research Council (NRC, 1996). The animals were given free access to food and tap water* ad libitum*.

### 2.7. Preparation of Rabbit Aorta

The vasorelaxant activity was carried out on rabbit aortic rings as per previous protocol [13]. Briefly, the animals were fasted for 24 h before experimentation. Rabbits were sacrificed by cervical dislocation, abdomen was cut, and aorta was removed from the thoracic of rabbit. Approximately, 2-3 mm of aorta was taken and suspended in an organ bath having 10 mL Krebs' solution. The temperature was maintained at 37°C and pH was adjusted to 7.4. Krebs' solution was composed of (mM) sodium chloride 118.2, glucose 11.7, magnesium sulphate 1.2, sodium bicarbonate 25.0, dihydrogen potassium phosphate 1.3, potassium chloride 4.7, and calcium chloride 2.5. In order to avoid anoxic condition, continuous supply of oxygen was maintained throughout experiment using carbogen (95% oxygen and 5% carbon dioxide). The suspended tissue was given a preload of 2 g and kept uninterrupted for 1 h as an equilibrium period. After that, the equilibrated tissues were treated with high K^+^ (80 mM) and phenylephrine (1 *μ*M) (PE) solutions to produce contractions. The effects of* R. indica* essential oil were tested against K^+^ and PE induced contraction in the tissue. Aorta preparations produced variations in the isometric tension in control and treated conditions were measured with force-displacement transducer (AD Instruments) coupled with PowerLab data acquisition system (AD Instruments, Sydney, Australia). The data were stored on computer and analyzed with LabChart 7 software (AD Instruments, Sydney, Australia). The data were plotted using GraphPad Prism version 5 software.

### 2.8. Preparation of Rabbit Jejunum

The smooth muscle relaxant or contracting activities of essential oil were evaluated using previously described methods [14]. In brief, the animals were kept abstained from food for 24 h before each experiment. The rabbits were sacrificed by cervical dislocation, the abdomen was cut open, and jejunum was isolated and freed from mesenteries and extra vascular tissues. The isolated jejunum of 2-3 cm was mounted on tissue bath containing 10 mL Tyrode's solution under continuous supply of oxygen. In current protocol, the composition of Tyrode's solution was in mM as KCl 2.7, NaCl 136.9, MgCl_2_ 1.1, NaHCO_3_ 11.9, NaH_2_PO_4_ 0.4, CaCl_2_ 1.8, and glucose 5.6. The temperature was set at 37°C and pH was adjusted to 7.4. A preload of 1 g was applied to the tissue and kept uninterrupted for 30 min. After stabilization, a submaximal dose of acetylcholine (Ach.) was used to achieve control responses. Subsequent to Ach.-induced reproducible responses,* R. indica* essential oil was applied in dose dependent manner. The effect produced by the essential oil was calculated as percent of control response produced by acetylcholine (Ach.). The isometric tension changes were measured using force transducer coupled with a bridge amplifier data acquisition system (AD Instruments, Sydney, Australia).

### 2.9. Calcium Channel Blocking Activity

The rabbit jejunum was used to find out the calcium channel blocking (CCB) effect of essential oil using well reported method [15]. The isolated tissue bath was added with K^+^ (80 mM) solution to depolarize the rabbit jejunum preparation. Essential oil of* R. indica* was applied in increasing order of dose after the formation of plateau to attain the concentration-dependent inhibitory curves. The calcium channel blocking effect of essential oil was measured as percent of control effect produced by high K^+^. After equilibration of jejunum preparation in Tyrode's solution, the tissues were washed with Ca^++^-free Tyrode's solution having Ethylenediaminetetraacetic Acid (EDTA). Hereafter, the calcium-free and potassium-rich Tyrode's solution was added to replace washing solution. After 30 min of tissue stabilization control curves (at least two cycles) were produced. The essential oil was treated in an increasing order of dose before producing a Ca^++^ curve to find out the calcium channel blocking activity. The calcium dose response curves (CDRCs) were reformed and compared with the control CDRC.

### 2.10. Statistical Analysis

Wherever applicable the statistical analysis was performed using GraphPad Prism version 5 software. The data given are expressed as ± standard error means (SEM), and the median effective concentrations (EC_50_ values) are given with 95% confidence intervals (CI).

## 3. Results

### 3.1. Chemical Analysis of the* R. indica* Essential Oil

**Figure 1:**
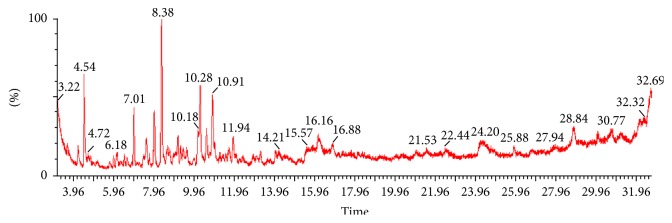
Gas chromatogram showing various components of essential oil obtained from fresh petals of* R. indica*.

The essential oil of* R. indica* was subjected to gas chromatography–mass spectrometry (GC-MS) analysis in order to determine its chemical composition. The GC chromatogram of* R. indica* essential oil is presented in Figure 1. The results showed that various chemical constituents are present in the essential oil of* R. indica*. The retention time (min), molecular mass, and formula for each identified component are mentioned in Table 1. The major constituents identified include acetic acid, mercaptohexyl ester, butanoic acid, 2-methyl-5-oxo-1-cyclopentene-1-yl ester, artemiseole, methyl santonilate, isosteviol, caryophyllene oxide, pentyl phenyl acetate, dihydromyrcene, 1,5-octadecadien,octadecanoic acid, ethyl ester, palmitic acid (2-phenyl-1,3-dioxolan-4-yl methyl ester), santolina epoxide, and 9-farnesene.

### 3.2. Vasorelaxant Activity of Essential Oil Derived from* R. indica*

**Figure 2:**
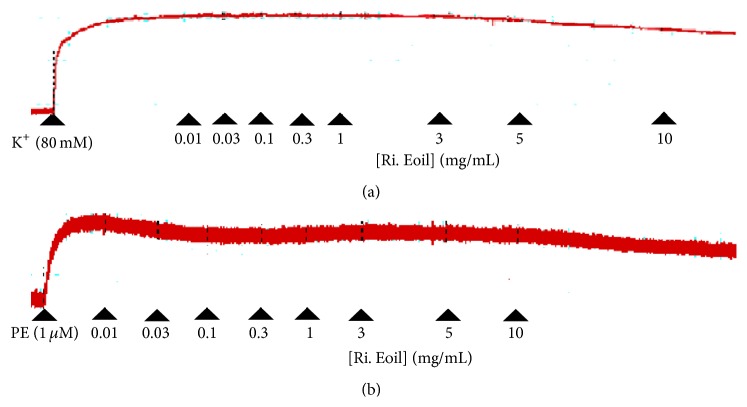
Typical tracing showing the effect of* R. indica* essential oil (Ri. Eoil) on (a) K^+^ (80 mM) and (b) phenylephrine (1 *μ*M) (PE) induced vasoconstrictions in isolated rabbit aorta preparations.

**Figure 3:**
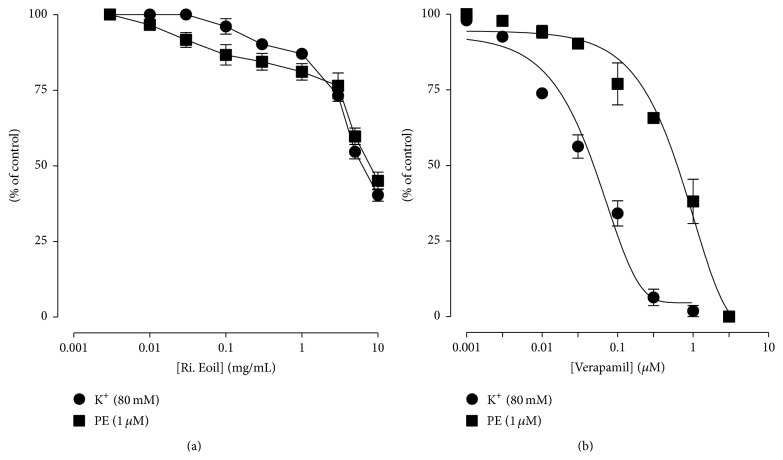
Typical graph shows the concentration-response curve of the essential oil from fresh petals of* R. indica* (Ri. Eoil) (a) and verapamil (1 *μ*M) (b) on K^+^ (80 mM) and phenylephrine (1 *μ*M) (PE) induced vasoconstrictions in isolated rabbit aorta preparations. Values shown are mean ± SEM (*n* = 3).

The vasorelaxant effect of the essential oil derived from fresh petals of* R. indica* was studied on aorta rings. The results showed that vasorelaxant effects were produced by the essential oil against PE and high K^+^. These results were compared with the vasorelaxant effect produced by verapamil against PE and K^+^ (80 mM) induced contractions (Figure 3(b)), thus showing vasorelaxant effect on rabbit aortic ring. As shown in Figures 2 and 3(a), the vasorelaxant effect of essential oil was started at 3 mg/mL against high K^+^ and 0.01 mg/mL for PE and the maximum effect was produced at 10 mg/mL against the contractions produced by both high K^+^ and PE. The median effective concentration (EC_50_) of essential oil for PE and high K^+^ is 7.39 mg/mL (5.0–9.78) and 5.80 (5.0–6.6). The median effective concentration (EC_50_) of verapamil is 0.58 mg/mL (0.3–0.86) and 0.0455 (0.03–0.061) on PE and high K^+^ induced contractions, respectively.

### 3.3. Spasmolytic Activity of Essential Oil Derived from* R. indica*

**Figure 4:**
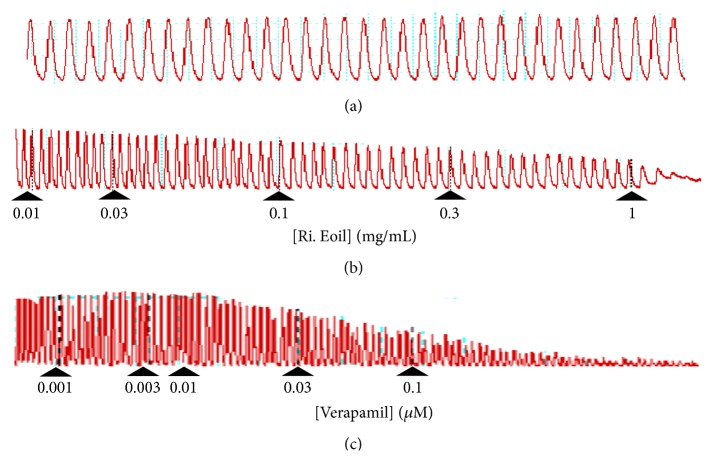
Typical tracing of the (a) normal contractions and relaxation, (b) concentration-dependent spasmolytic effect of essential oil isolated from* Rosa indica* (Ri. Eoil), and (c) verapamil on spontaneously contracted isolated rabbit jejunum preparations.

**Figure 5:**
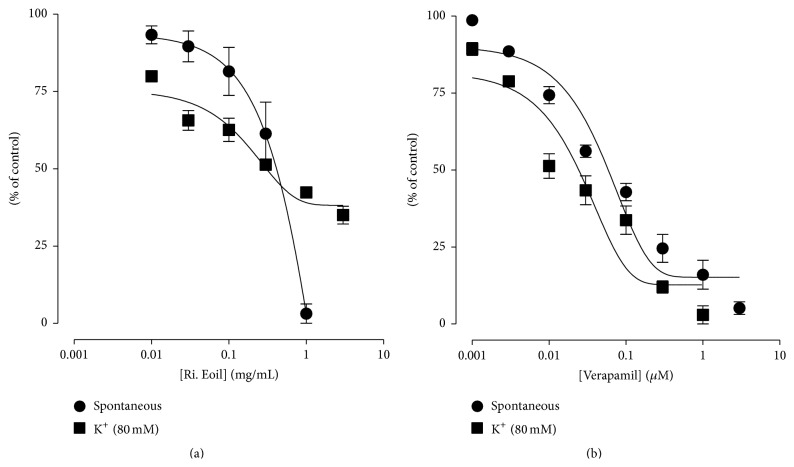
Graph showing (a) the concentration-dependent spasmolytic effect of* R. indica* essential oil (Ri. Eoil) and (b) verapamil (1 *μ*M) against spontaneous contractions and contraction induced by K^+^ (80 mM) in isolated jejunum preparation. Values shown are mean ± SEM (*n* = 3).

The spasmolytic effect of essential oil derived from* R. indica* was measured at different dose levels in cumulative manner (0.01–1 mg/mL) using isolated jejunum of rabbit. The effect was started at 0.01 mg/mL against spontaneous contractions and tissue was completely relaxed at 1.0 mg/mL dose (Figures 4(b) and 5(a)). Normal contractions and relaxation (spontaneous contraction) are shown in Figure 4(a). The calculated (*n* = 3) effective concentration (EC_50_) of essential oil for spontaneous and high K^+^ was 0.418 mg/mL (0.3–0.536) and 0.298 (0.1–0.496), respectively. The spasmolytic effect for the essential oil (Figure 4(b)) was also compared with verapamil (standard drug) (Figures 4(c) and 5(b)). The effective concentration (EC_50_) of verapamil against spontaneous and high K^+^ was 0.0539 mg/mL (0.03–0.0778) and 0.0236 (0.01–0.0372), respectively. The comparative results showed that essential oil has verapamil-like smooth muscle relaxing or spasmolytic activity.

### 3.4. Calcium Channel Blocking Activity of* R. indica* Essential Oil

**Figure 6:**
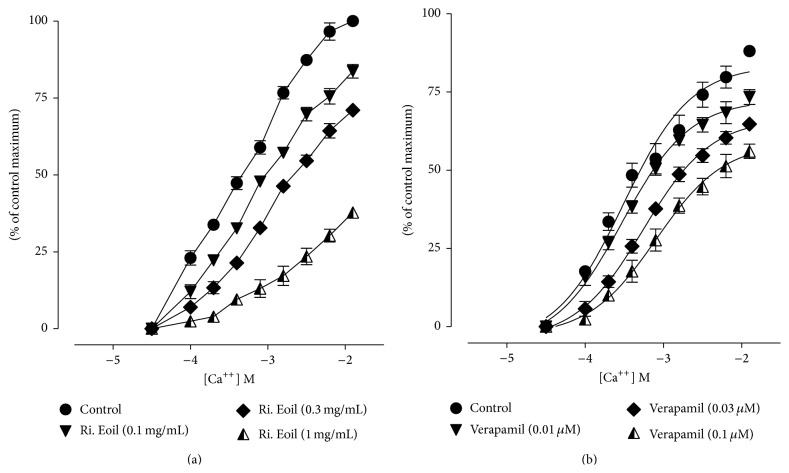
Concentration-response curves of calcium showing the spasmolytic effect of (a)* R. indica* essential oil (Ri. Eoil) and (b) verapamil. Values shown are mean ± SEM (*n* = 3).

To find out the mechanism of action for* R. indica* essential oil spasmolytic effects, the calcium channel blocking activity was measured on isolated jejunum. For this purpose, 0.1–1 mg/mL doses of essential oil were applied on tissue to obtain concentration-dependent response curves for calcium. It was observed that* R. indica* essential oil has the ability to block calcium channels (Figure 6(a)). As shown in Figures 6(a) and 6(b) the essential oil and verapamil (reference drug) caused rightward shift of calcium curves which shows the channel blocking activity of essential oil.

## 4. Discussion

*R. indica* is traditionally used in heart and gut disorders [16].* R. damascena* essential oil has been reported for antispasmodic uses in folkloric medicine [6]. However, current literature lacks the scientific pharmacological investigation of the* R. indica* in such diseases. It is also important for scientific investigation to know the chemical constituents of used products. Therefore, the current study was undertaken to scientifically evaluate the potentials of* R. indica* essential oil as an alternative medicine in cardiovascular and gut diseases. Investigation of the chemical component of the essential oil was also aimed for in this study.

GC-MS is the most widely used technique for the identification of chemical composition essential oils, therefore;* R. indica* essential oil was analyzed through this method. The major constituents identified in the essential oil were methyl santonilate, butanoic acid, 2-methyl-5-oxo-1-cyclopentene-1-yl ester, santolina epoxide, artemiseole, 9-farnesene, octadecanoic acid ethyl ester, palmitic acid (2-phenyl-1,3-dioxolan-4-yl methyl ester), isosteviol, caryophylline oxide, pentyl phenyl acetate, and dihydromyrcene (Figure 1; Table 1). In literature, caryophyllene oxide was also reported from shade-dried petals of* R. damascene* [17]. Similarly, farnesene and caryophyllene were reported in* R. hybrida* floral fragrances [18]. Some peaks of GC were unidentified however; several new compounds including methyl santonilate, butanoic acid, 2-methyl-5-oxo-1-cyclopentene-1-yl ester, santolina epoxide, artemiseole, octadecanoic acid ethyl ester, palmitic acid (2-phenyl-1,3-dioxolan-4-yl methyl ester), isosteviol, pentyl phenyl acetate, and dihydromyrcene were detected in the essential oil of* R. indica*. Some of the idenfided compounds have been reported to possess various biological activities including postive effects in cardiovascular and gastrointestinal disorders. The major component, *β*-caryophyllene (sesquiterpene), is considered as a calcium channel blocker and produces significant reduction in blood pressure [19]. Isosteviol (a component of* R. indica* essential oil) has also been reported to have a vasodilator activity [20].

Calcium plays an important role in the normal function of cells. There is a concentration gradient of calcium across the cell membrane. Changes in this concentration gradient have a vital role in contracting and relaxing process of vascular smooth muscle (VSM) cells [21]. Calcium mainly moves (in or out) across the plasma membrane through specialized calcium channels. The sudden opening or closure of these channels disturbs the normal concentration gradient and hence the cell membrane undergoes depolarization. Cell membrane depolarization produces a structural variation in the calcium channel which allows the outside calcium to get into the cell. In VSM cells, the calcium entry into the cell causes the internal calcium mobilization through sarcoplasmic stores that lead to actin-myosin complex activation and thus a muscular contraction occurs [22]. These calcium channels are divided into different types and subtypes. L-types, “long-acting,” are the voltage-sensitive calcium channels that are distributed throughout the cardiovascular (CVS) system. It was reported that changes in L-type channels have an important role in hypertension [23]. The currently available CCBs normally target L-type calcium channels for the treatment of hypertension [24]. The main molecular mechanism of action for CCBs is that it blocks the calcium channels through binding to sites that are responsible for calcium channels opening. Therefore, CCBs interfere with calcium influx and inhibit membrane depolarization and VSM cell contraction (vasorelaxant).

In the current study,* R. indica* essential oil was tested for vasorelaxant activity where dose dependent effects were observed (Figure 2) against the PE precontracted tissues. It is important to mention that maximum relaxation was observed at 10 mg/mL. Similarly,* R. indica* essential oil also produced tissue relaxation against high K^+^ (80 mM) precontracted tissues (Figure 2).* R. indica* was found more potent against K^+^ (80 mM) induced contractions as compared to PE (1 *μ*M) (Figure 3). Agents that relax PE contracted tissues are considered to be inhibitor of internal calcium mobilization. On the other hand, substances inhibiting K^+^ (80 mM) produced contractions are recognized as CCBs [25]. Therefore, the above mentioned results suggested that* R. indica* essential oil is more potent CCBs rather than inhibitor of internal calcium mobilization. CCBs are beneficial in the treatment of hypertension [26]; thus* R. indica* essential oil may be used as an antihypertensive agent.

In an isolated rabbit jejunum preparation,* R. indica* essential oil was studied for spasmolytic activity and verapamil were used as standard drug. The essential oil produced concentration-dependent relaxation in spontaneous contractions (Figure 4(b)) as well as contraction induced by high K^+^ (Figure 5(a)). The relaxation (spasmolytic) pattern of the intestinal smooth muscle caused by the essential oil was similar to the standard drug (Figures 4(c) and 5(b)). Calcium plays crucial role in the normal functions of gastrointestinal smooth muscle cells. Increase in calcium influx causes disease conditions like diarrhea and spasms in gastrointestinal tract. So calcium channel blockers are considered to be effective in diarrhea and gut spasms [27]. Therefore, essential oil of* R. indica* may act as antidiarrheal and antispasmodic agent. The spasmolytic activity for any essential oil of specie of the genus* Rosa* has not been reported. It is reported in literature that agents having spasmolytic activity mediate their relaxant effects through calcium channel blockade (CCB) [28]. The treatment of high K^+^ caused depolarization in isolated rabbit jejunum that led to an increase in free calcium level of cytoplasm which ultimately produced contractions in the smooth muscle [29]. The increase in calcium level of cytoplasm was either through an influx of calcium via voltage dependent channels (VDCs) or through release of calcium from intracellular stores. It was reported that high K^+^ treatments produce depolarization by an influx of calcium through VDCs [30]. Therefore, the relaxation of essential oil against high K^+^ contraction suggests that* R. indica* has the potential to block VDCs. The calcium channel blocking effect of the essential oil was further verified when the* R. indica* essential oil pretreatment caused a rightward shift in Ca^++^ curves (Figure 6(a)) similarly to verapamil (Figure 6(b)). This shifting of calcium curves to the right is showing relaxation of the tissue [15]. The essential oil produced the spasmolytic effect (Figure 6(a)) in a similar fashion as observed for the standard drug (Figure 6(b)). The verapamil belongs to phenyl alkyl amine class of CCBs. Thus the relaxing effect of* R. indica* on rabbit jejunum may be due to blocking of calcium influx via VDCs; hence it work as CCBs.

## 5. Conclusion

Based on the results obtained it can be concluded that several compounds are present in the essential oil of* R. indica*. However, the correct identification of compounds based only on library search is not enough; therefore, more detailed chemical analyses of the* R. indica* essential oils are suggested in future studies. Moreover, the essential oil has vasorelaxant activity. Similarly,* R. indica* showed an excellent spasmolytic activity. It is important to mention that essential oil showed calcium channel blockade-like mechanism. This study provided scientific evidences that* R. indica* essential oil has great potential to be used for the remedies of various cardiovascular and gastrointestinal disorders especially hypertension and gut spasm.

## Figures and Tables

**Table 1:** Components of *R*. *indica* essential oil identified through GC-MS analysis.

Component	Ret. time (min)	Mol. Wt.	Mol. formula
Acetic acid, mercaptohexyl ester	4.72	232	C_12_H_24_O_2_S
Butanoic acid, 2-methyl-5-oxo-1-cyclopentene-1-yl ester	4.54	182	C_10_H_14_O_3_
Artemiseole	28.84	152	C_10_H_16_O
Methyl santonilate	7.01	182	C_11_H_18_O_2_
Isosteviol	10.28	318	C_20_H_30_O_3_
Caryophyllene oxide	10.91	220	C_15_H_24_O
Pentyl phenyl acetate	16.16	206	C_13_H_18_O_2_
Dihydromyrcene	34.04	138	C_10_H_18_
1,5-Octadecadien	49.54	134	C_10_H_14_
Octadecanoic acid, ethyl ester	48.09	312	C_20_H_40_O_2_
Palmitic acid (2-phenyl-1,3-dioxolan-4-yl methyl ester)	48.12	418	C_26_H_42_O_4_
Santolina epoxide	8.38	152	C_10_H_16_O
9-Farnesene	39.25	204	C_15_H_24_

## References

[1] Crepet W. L., Nixon K. C., Gandolfo M. A. (2004). Fossil evidence and phylogeny: the age of major angiosperm clades based on mesofossil and macrofossil evidence from cretaceous deposits. *American Journal of Botany*.

[2] Judd W., Campbell C., Kellogg E., Stevens P., Donoghue M. (1999). *Plant Systematics. A Phylogenetic Approach*.

[3] Farooq A., Khan M. A., Ali A., Riaz A. (2011). Diversity of morphology and oil content of *Rosa damascena* landraces and related Rosa species from pakistan. *Pakistan Journal of Agricultural Sciences*.

[4] Hunt S. R. (1962). The rose in pharmacy. *The Pharmaceutical Journal*.

[5] Singh V., Kaul V. K., Singh B., Sood R. P., Handa S. S., Kaul M. K. (1997). Damask rose (*Rosa damascena* Mill.): cultivation and processing. *Supplement to Cultivation & Utilization of Aromatic Plants*.

[6] Sadraei H., Asghari G., Emami S. (2013). Inhibitory effect of *Rosa damascena* Mill flower essential oil, geraniol and citronellol on rat ileum contraction. *Research in Pharmaceutical Sciences*.

[7] Basim E., Basim H. (2003). Antibacterial activity of *Rosa damascena* essential oil. *Fitoterapia*.

[8] Karthy E. S., Ranjitha P., Mohankumar A. (2009). Antimicrobial potential of plant seed extracts against multidrug resistant methicillin resistant Staphylococcus aureus (MDR-MRSA). *International Journal of Biology*.

[9] Boskabady M. H., Shafei M. N., Saberi Z., Amini S. (2011). Pharmacological effects of *Rosa damascena*. *Iranian Journal of Basic Medical Sciences*.

[10] Bai S., Seasotiya L., Malik A., Bharti P., Dalal S. (2015). Bioactive compounds and pharmacological potential of *Rosa indica* L. and *Psidium guajava* L. methanol extracts as antiurease and anticollagenase agents. *Der Pharmacia Lettre*.

[11] Verma R. S., Padalia R. C., Chauhan A., Singh A., Yadav A. K. (2011). Volatile constituents of essential oil and rose water of damask rose (*Rosa damascena* mill.) cultivars from north indian hills. *Natural Product Research*.

[12] Lima B. G., Tietbohl L. A. C., Fernandes C. P. (2012). Chemical composition of essential oils and anticholinesterasic activity of *Eugenia sulcata* spring ex mart. *Latin American Journal of Pharmacy*.

[13] Shah A. J., Gilani A. H. (2009). Blood pressure-lowering and vascular modulator effects of *Acorus calamus* extract are mediated through multiple pathways. *Journal of Cardiovascular Pharmacology*.

[14] Shah A. J., Bhulani N. N., Khan S. H., Rehman N. U., Gilani A. H. (2010). Calcium channel blocking activity of *Mentha longifolia* L. explains its medicinal use in diarrhoea and gut spasm. *Phytotherapy Research*.

[15] Shah A. J., Zaidi M. A., Sajjad H., Hamidullah, Gilani A.-H. (2011). Antidiarrheal and antispasmodic activities of *Vincetoxicum stocksii* are mediated through calcium channel blockade. *Bangladesh Journal of Pharmacology*.

[16] Schweisheimer W. (1961). Roses in manufacture of perfumes. *Perfums Cosmetics and Savons*.

[17] Verma R. S., Padalia R. C., Chauhan A. (2011). Chemical investigation of the volatile components of shade-dried petals of damask rose (*Rosa damascena* Mill.). *Archives of Biological Sciences*.

[18] Kim H.-J., Kim K., Kim N.-S., Lee D.-S. (2000). Determination of floral fragrances of *Rosa hybrida* using solid-phase trapping-solvent extraction and gas chromatography-mass spectrometry. *Journal of Chromatography A*.

[19] Bigliani M. C., Rossetti V., Grondona E. (2012). Chemical compositions and properties of *Schinus areira* L. essential oil on airway inflammation and cardiovascular system of mice and rabbits. *Food and Chemical Toxicology*.

[20] Wong K.-L., Chan P., Yang H.-Y. (2004). Isosteviol acts on potassium channels to relax isolated aortic strips of Wistar rat. *Life Sciences*.

[21] Katz A. M. (1997). Molecular biology of calcium channels in the cardiovascular system. *The American Journal of Cardiology*.

[22] Fleckenstein-Grun G. (1996). Calcium antagonism in vascular smooth muscle cells. *Pflugers Archiv European Journal of Physiology*.

[23] Spedding M., Paoletti R. (1992). Classification of calcium channels and the sites of action of drugs modifying channel function. *Pharmacological Reviews*.

[24] Triggle D. J. (1998). The physiological and pharmacological significance of cardiovascular t-type, voltage-gated calcium channels. *American Journal of Hypertension*.

[25] Godfraind T., Miller R., Wibo M. (1986). Calcium antagonism and calcium entry blockade. *Pharmacological Reviews*.

[26] Brunton L. L., Hardman J. G., Limbird L. E., Molinoff P. B. (1996). Agents affecting gastrointestinal water flux and motility; emesis and antiemetics; bile acids and pancreatic enzymes. *Goodman and Gillman's the Pharmacological Basis of Therapeutics*.

[27] Ogilvie R. I., Burgess E. D., Cusson J. R., Feldman R. D., Leiter L. A., Myers M. G. (1993). Report of the Canadian hypertension society consensus conference: 3. Pharmacologic treatment of essential hypertension. *Canadian Medical Association Journal*.

[28] Gilani A. U. H., Shah A. J., Ahmad M., Shaheen F. (2006). Antispasmodic effect of *Acorus calamus* Linn. is mediated through calcium channel blockade. *Phytotherapy Research*.

[29] Karaki H., Weiss G. B. (1988). Calcium release in smooth muscle. *Life Sciences*.

[30] Brading A. F. (1981). How do drugs initiate contraction in smooth muscles?. *Trends in Pharmacological Sciences*.

